# Medicated Milk

**Published:** 1847-01

**Authors:** 


*Medicated Milk.*—The Editor of Gazette Medicale (for June, 1846,) mentions that there has been lately established at Montrouge, near Paris, an establishment of a novel kind. The physicians who superintend it, thought proper to treat certain diseases by the use of cows' and goats' milk, having first subjected these animals to the medication necessary to give their milk the therapeutic properties which may be required for the cure of these diseases.—*Southern Med. Jour.*

